# Alveolar Bone Microstructure Surrounding Orthodontic Anchor Screws with Plasma Surface Treatment in Rats

**DOI:** 10.3390/jfb14070356

**Published:** 2023-07-07

**Authors:** Keisuke Okawa, Satoru Matsunaga, Norio Kasahara, Masaaki Kasahara, Chie Tachiki, Takayoshi Nakano, Shinichi Abe, Yasushi Nishii

**Affiliations:** 1 Department of Orthodontics, Tokyo Dental College, 2-9-18 Kandamisaki-cho, Chiyoda-ku, Tokyo 101-0061, Japan; tyuujoukeisuke@tdc.ac.jp (K.O.); tachikichie@tdc.ac.jp (C.T.); nishii@tdc.ac.jp (Y.N.); 2 Oral Health Science Center, Tokyo Dental College, 2-9-18 Kandamisaki-cho, Chiyoda-ku, Tokyo 101-0061, Japan; nkasahara@tdc.ac.jp (N.K.); kasaharamasaaki@tdc.ac.jp (M.K.); abesh@tdc.ac.jp (S.A.); 3 Department of Anatomy, Tokyo Dental College, 2-9-18 Kandamisaki-cho, Chiyoda-ku, Tokyo 101-0061, Japan; 4 Department of Histology and Developmental Biology, Tokyo Dental College, 2-9-18 Kandamisaki-cho, Chiyoda-ku, Tokyo 101-0061, Japan; 5 Department of Dental Materials Science, Tokyo Dental College, 2-9-18 Kandamisaki-cho, Chiyoda-ku, Tokyo 101-0061, Japan; 6 Division of Materials and Manufacturing Science, Graduate School of Engineering, Osaka University, 2-1, Yamadaoka, Suita 565-0871, Japan; nakano@mat.eng.osaka-u.ac.jp

**Keywords:** bone quality, biological apatite orientation, collagen fiber, miniscrews, horizontal loading

## Abstract

A lateral load was applied to anchor screws that had undergone surface treatment, and the structure, cellular dynamics, and quality of the bone surrounding anchor screws were analyzed to investigate the effect of this surface treatment on the peri-implant jawbone. In addition, bone microstructural characteristics were quantitatively evaluated for each site of loading on the bone around the anchor screw. Rats were euthanized after observation on days 3, 5, or 7, and bone quality analyses were performed. Bone–implant contact rate increased more rapidly at an early stage in the treated surface group than in the untreated surface group. Bone lacuna morphometry showed that the measured values adjacent to the screw at the screw neck on the compressed side (A) and at the screw tip on the uncompressed side (D) were significantly lower than those at the screw tip on the compressed side (B) and at the screw neck on the uncompressed side (C). Collagen fiber bundle diameter showed that the measured values adjacent to regions A and D were significantly higher than those at regions B and C. Anchor screw surface activation facilitates initial bone contact of the screw, suggesting that early loading may be possible in clinical practice.

## 1. Introduction

In recent years, the use of orthodontic anchor screws (hereafter referred to as anchor screws) has become common in the orthodontic treatment of dental and skeletal malocclusions to provide a stable fixation source [1,2,3]. In particular, the use of anchor screws is now being actively used in orthodontic treatment, as the conventional orthodontic treatment of centrifugal movement or pressure reduction of molars does not provide sufficient tooth movement [4,5]. However, Reynders et al. reported that the success rate of anchor screw retention during orthodontic treatment is 80–90%, and they have a comparatively higher rate of anchor screw loss than dental implants, with a reported success rate of 96.7–100% [6,7,8]. Causes of anchor screw loss include oral hygiene, bone density, cortical bone thickness, and the load applied to the anchor screw [9].

Studies of dental implants have shown that stability is improved by early osseointegration [10,11]. Research focusing on the surface properties of the implant includes a study by Khan et al. that showed that initial osteoblast adhesion and fibronectin adsorption are improved by a hydrophilic surface [12]. Mo et al. also reported that treating the implant surface to make it hydrophilic significantly increased the removal torque values, suggesting that surface treatment might be effective [10]. Yoshinari et al. reported that the effects of plasma surface treatment are the decomposition of hydrophobic organic matter (hydrocarbons) adsorbed on the surface and the formation of more hydroxyl groups (-OH) on the surface by reacting with hydrocarbon molecules adsorbed on the surface and hydrogen of water molecules in the atmosphere. As a result, the effect of the decomposition of hydrophobic organic matter (hydrocarbons) adsorbed on the surface and the introduction of hydroxyl groups on the titanium dioxide surface combine to produce super-hydrophilic properties [13]. Among a variety of surface treatment techniques, including sandblasting, etching, and UV treatment, plasma surface treatment was chosen. Plasma surface treatment may be finished quickly at the chairside at atmospheric pressure and is anticipated to have clinical applications. Although bacterial infection and overload are frequently stated as reasons for implant loss, it is challenging to directly assess the mechanical function of the peri-implant bone, and numerous unresolved inquiries exist.

Bone mineral density (BMD) is the main index that has been conventionally used to evaluate the mechanical function of peri-implant bone [14,15]. However, it is common to encounter patients with fractures despite their having a high density, and it is now clear that a number of other factors are also implicated in bone mechanical function [16]. Since the NIH Consensus Conference in 2000, which established bone density as a factor implicated in bone mechanical performance, it has become widely recognized that bone quality, in addition to bone density, also plays a significant biomechanical role [17]. Nakano et al. investigated such bone quality factors as bioapatite (BAp) crystal orientation and collagen fiber anisotropy and found that bone quality has a strong correlation with bone mechanical function [18]. Therefore, they reported that it is possible to predict the mechanical function of the jawbone by searching for running anisotropy of collagen fibers and orientation of BAp crystals, which are bone quality factors.

The application of an excessive lateral load to dental implants causes peri-implant bone resorption, and it should be therefore performed with caution [19]. Anchor screws, unlike dental implants, are used as reinforced anchorage in orthodontic treatment [20]. This means that the main load applied to the bone surrounding anchor screws is an excessive lateral load, but much concerning the effect of this change in the mechanical environment of the bone around these screws remains unclear. Matsumoto et al. reported that the quality of the bone around the screws changed in response to lateral loading [21]. However, the research on anchor screws and lateral loading cannot be described as sufficient, and a few studies have looked at whether surface treatment of anchor screws is effective in enabling them to fulfill their function as anchorage. In-depth investigations of the detailed bone dynamics of the bone surrounding anchor screws and their effect on bone density and quality are, therefore, an urgent task.

In this study, a lateral load was applied to anchor screws that had undergone surface treatment, and the structure, cellular dynamics, and quality of the bone surrounding anchor screws were analyzed to investigate the effect of this surface treatment on the peri-implant jawbone. In addition, bone microstructural characteristics of each region were quantitatively evaluated by loading applied to the bone surrounding the anchor screw.

## 2. Materials and Methods

### 2.1. Specimens

Adult male Wister rats (25 week-old; average weight 400 g; 24 animals) were used in the animal experiments. Rats were housed under a 12h light/dark cycle with ad libitum access to food and water. This study was conducted under the approval of the Ethics Review Committee for Animal Experiments of Tokyo Dental College (Ethics Application No. 213104).

### 2.2. Anchor Screw Surface Treatment

**Figure 1:**
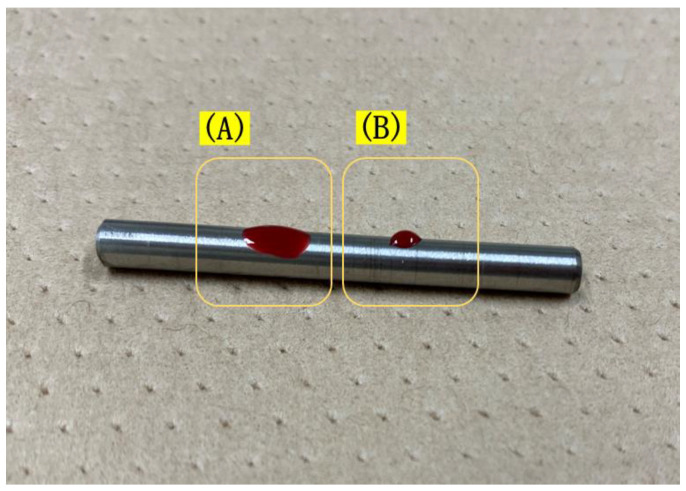
Effects of plasma surface treatment. Image of a drop of rat blood on a titanium alloy of the same composition as the orthodontist’s anchor screw. (**A**) is the area where plasma surface treatment was performed. (**B**) is untreated area.

Half of the anchor screws (Le Forte System, Ti-6Al-4V titanium alloy, diameter 1.2 mm, length 3 mm; Pro-Seed, Tokyo, Japan) were treated by plasma surface treatment with a PZ2 piezo brush^®^ (Relyon Plasma, Regensburg, Germany) for 30 s, and half were left untreated (Figure 1). Anchor screw surfaces were treated right before implantation. Those screws that underwent plasma surface treatment and on which a load was imposed were designated the plasma–treated loading (PTL) group, and those that underwent plasma surface treatment but on which no load was imposed were the plasma–treated unloaded (PTU) group. As control groups, those anchor screws that had not undergone plasma treatment and to which a load was applied were designated the surface–untreated loaded (SUL) group, and those that had not undergone plasma treatment and to which no load was applied the surface untreated unloaded (SUU) group.

### 2.3. Experimental Procedure

**Figure 2:**
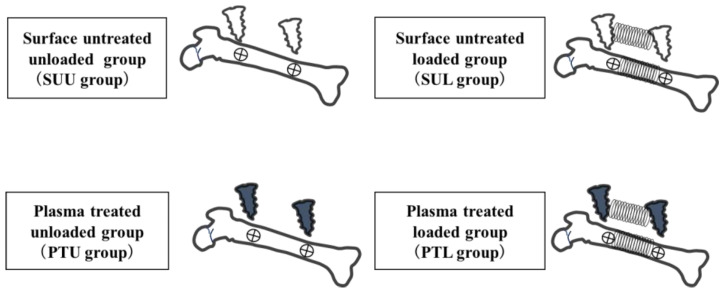
Experimental protocol. The control groups comprised anchor screws to which no lateral load was applied that had not undergone plasma treatment (surface–untreated unloaded (SUU) group) and that had undergone plasma treatment (plasma–treated unloaded (PTU) group). In the experimental groups, two screws in the left femur that had not undergone surface treatment were connected with a Ni-Ti coil spring to apply a lateral load (0.5 N) (surface–untreated loaded (SUL) group), as were two screws that had undergone surface treatment (plasma–treated loaded (PTL) group). Experimental specimens were harvested after a healing period of 3, 5, or 7 days.

**Figure 3:**
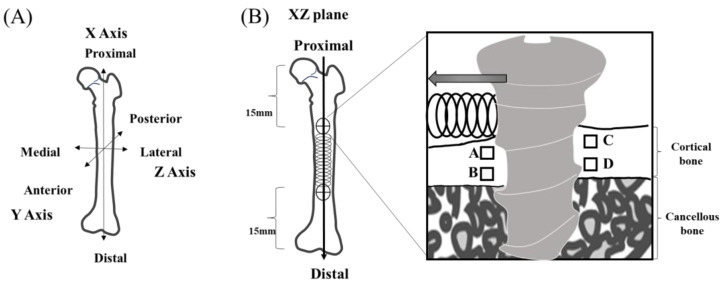
Axis definitions. (**A**) The X-axis is defined as the long axis of the femur, the Y-axis as the anterior–posterior direction, and the Z-axis as the medial–lateral direction. (**B**) Region of interest (ROI) definitions. ROIs are defined as 100 µm × 100 µm squares parallel to the X-axis at distances of 50 µm from the surface layer of cortical bone and the cortical–cancellous boundary and a distance of 100 µm from the screw body. An anchor screw was inserted above the periosteum in the direction of the Y-axis at the anterior margin of the proximal diaphyseal region (15 mm from the hip joint) and the anterior margin of the distal diaphyseal region (15 mm from the knee joint) at a site.

The 24 adult Wistar rats were randomly allocated to a control group (the unloaded group) and an experimental group (the loaded group) (Figure 2). A three-axis system was set up as the reference axis for the samples, with the X-axis in the direction of the long axis of the rat femur, the Y-axis in the anteroposterior direction, and the Z-axis in the medial and posterolateral directions (Figure 3). After habituation for one week, implantation was executed. Anchor screw placement was performed under general anesthesia with intraperitoneal administration of a triad of anesthetics (0.75 mg/kg medetomidine hydrochloride, Nippon Zenyaku Kogyo, Fukushima, Japan; 4.0 mg/kg, midazolam, Sand Corporation, Tokyo, Japan; 5.0 mg/kg, butorphanol tartrate, Meiji Seika Pharma Corporation, Tokyo, Japan). The skin above the left femur was incised about 20 mm from the ventral side of the rat; the muscle layer was separated and developed, and the femur was clarified. After peeling, preparatory drilling was performed in the Y-axis direction using a drill (0.5 mm and 0.8 mm in diameter, Tamiya Co., Ltd., Shizuoka, Japan) for an underwater injection. Anchor screws were placed in the Y-axis direction on the periosteum at the anterior margin of the bilateral proximal femoral diaphysis (15 mm from the hip joint) and the anterior margin of the distal diaphysis (15 mm from the knee joint) where there was no muscle attachment in each case. A Ni-Ti coil spring (Dentos NT Coil Spring NT20-13U, Shofu, Kyoto, Japan) wrapped with cellophane tape to prevent tissue wandering was attached to the anchor screw on the left femur, and a 0.5 N lateral load was applied [22]. No load was imposed on the anchor screws in the right femur. The muscle layer and skin were then sutured with 5-0 nylon thread, and a medetomidine antagonist (0.75 mg/kg, atipamezole hydrochloride, Nippon Zenyaku Kogyo, Fukushima, Japan) was administered intraperitoneally immediately after the surgery to maintain the rats’ body temperature. The rats in each group were then euthanized on day 3, day 5, or day 7 after anchor screw implantation, and both femurs were harvested as experimental specimens (*n* = 4 in each group). After harvesting, each femur was fixed by immersion in 10% buffered formalin at 4 °C for 2 days. All femurs were scanned by micro-computed tomography (µCT-50, Scanco Medical AG, Wangen-Brüttisellen, Switzerland), and bone specimens containing both anchor screws were harvested after binding between the anchor screws, and the femur had been confirmed.

### 2.4. Micro-CT Scanning

All samples were scanned by micro-CT (μCT50, Scanco Medical AG, Wangen-Brüttisellen, Switzerland), and three-dimensional (3D) images were constructed by volume rendering with TRI/3D-BON-FCS64 analysis software (Ratoc System Engineering, Tokyo, Japan). The scanning conditions were as follows: tube voltage 90 kV; tube current 155 μA; image matrix 3400 × 3400; and slice thickness 2 μm.

### 2.5. Regions of Interest

Four regions of interest (ROIs) were designated with reference to the previous study by Matsumoto et al. [20]. Region A was a 100 µm × 100 µm square parallel to the X-axis located 50 µm from the cortical bone surface layer and 100 µm from the screw body on the compressed side, and Region B was a 100 µm square parallel to the X-axis located 50 µm from the cortical–cancellous boundary and 100 µm from the screw body on the same side. Regions C and D were similarly designated with reference to the cortical bone surface layer and the cortical–cancellous boundary on the uncompressed side (Figure 2).

### 2.6. Bone–Implant Contact (BIC)

The bone–implant contact rate (BIC, %) was calculated as the area of bone-to-implant contact as a proportion of the external circumference of the implant body in each ROI by analyzing the micro-CT images with image processing software (TRI/3D-BON-FCS64).

### 2.7. Bone Mineral Density (BMD)

In each group, BMD (mgHA/cm^3^) of the peri-implant bone was measured in each ROI using micro-CT analysis (Scanco Medical AG, Wangen-Brüttisellen, Switzerland). Five points were chosen at random within each ROI; the BMD of each was measured, and the mean value was calculated.

### 2.8. Histological Evaluation

The specimens were pre-stained using stepwise ethanol dehydration and Villanueva Osteochrome Bone Stain (Funakoshi Corporation, Tokyo, Japan), embedded in unsaturated polyester resin (Ligolac, Nisshin EM Corporation, Tokyo, Japan), and then sliced with a rotary microtome (SP1600, Leica, Nussloch, Germany) in the XY plane through the center of the two anchor screws. Next, polishing was performed using water-resistant abrasive paper (#400 to #800 and #1200) to prepare 100 µm thin-sliced polished specimens. The obtained samples were observed with a universal optical microscope (Axiophot2; Carl Zeiss, Oberkochen, Germany), and the morphology of each ROI around the anchor screw was analyzed with the supplied imaging software; Axiovision 4.8.2 (Carl Zeiss, Oberkochen, Germany). The morphology of the bone lacunas at each ROI around the anchor screw was analyzed. In bone lacuna morphometry measurements, the ratio of the long diameter of the bone lacunas at the site at which their width was the greatest and the corresponding short diameter measured on a line perpendicular to the center of the long diameter was calculated for each group.

### 2.9. Second Harmonic Generation Imaging (SHG Imaging)

Second harmonic generation (SHG) images were obtained using a multiphoton confocal microscopy system (LSM 880 Airy NLO; Carl Zeiss, Oberkochen, Germany) with an excitation laser (Chameleon Vision II, wavelengths: 680-1080 nm; repetition rate: 80 MHz; pulse width: 140 fs; Coherent Inc. Apochromat 10×/0.8 M27; Carl Zeiss, Oberkochen, Germany). The excitation wavelength for observation of collagen fibers was 880 nm. Software (ZEN, Carl Zeiss, Oberkochen, Germany) was used for image acquisition. After image acquisition, the collagen fiber bundles in the calcified area of interest were traced using Imaris 8.4 (Bitplane AG, Switzerland), and the fiber bundle diameters were measured.

### 2.10. BAp Crystal Orientation

Quantitative evaluation of BAp crystal orientation was performed using a curved imaging plate X-ray diffractometer with optics (XRD, D/MAX RAPIDII-CMF, Rigaku Corporation, Tokyo, Japan), using an optical microscope (0.6–4.8×) attached to the XRD, with the irradiated area positioned at the center of the cortical bone. X-rays were irradiated so that the incident beam was a circle with a diameter of 100 μm. The position of the specimen was determined using the X-, Y-, and Z-axes as reference axes. Measurements were taken at the ROI. The measurements were performed in the ROIs. Measurements were performed according to Nakano et al. [18] using a transmission optics system and a reflection optics system, and Cu-Kα radiation was used as the source of the radiation. The setup conditions were a tube voltage of 40 kV and a tube current of 30 mA.

From the diffraction ring image drawn on the imaging plate by diffracted X-rays, the X-ray intensity ratio of the two diffraction peaks in the (002) and (310) planes was calculated using 2D Data Processing Software ver. 2.1.6 (Rigaku Corporation, Tokyo, Japan).

### 2.11. Statistical Analysis

Statistical analysis was performed using Tukey’s test for the mean of each group. The *p*-value to be considered statistically significant was less than 0.05.

## 3. Results

**Figure 4:**
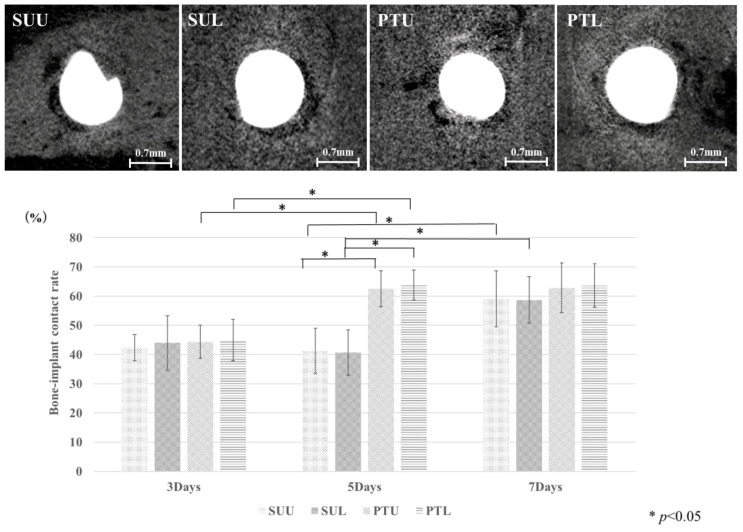
Images of micro-CT of each group at 5 days and BIC measurement. The vertical axis is BIC %. The BIC increases at an earlier stage in the surface treatment group.

**Figure 5:**
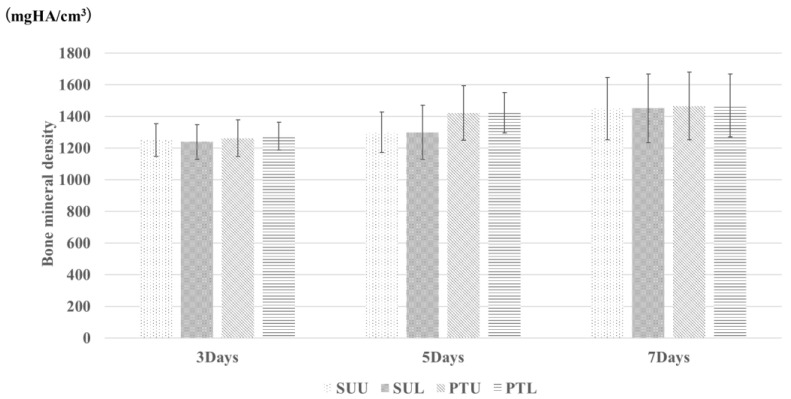
BMD. The vertical axis is BMD mmHg/cm^3^. This tends to increase over time, but the difference is not significant.

Figure 4 shows micro-CT images of each group at 5 days and the measured BIC rates for the SUL, SUU, PTL, and PTU groups. In the PTL and PTU groups, the measured value was significantly higher on day 5 than on day 3. In the SUL and SUU groups, the measured value was significantly higher on day 7 than on day 5. The measured values on day 5 were significantly higher in the PTL and PTU groups than in the SUL and SUU groups. There were no significant differences between the loaded and unloaded groups. Figure 5 shows the measured BMD values in the SUL, SUU, PTL, and PTU groups. Although these tended to increase in all the groups, the difference was not significant in any case.

**Figure 6:**
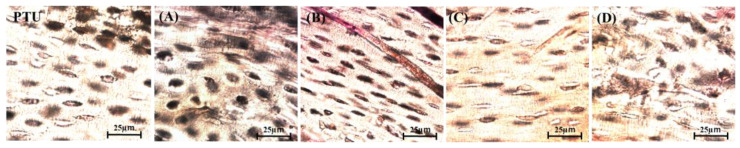
Villanueva staining. PTL group, region A (**A**). PTL group, region B (**B**), magnified image. PTL group, region C, magnified image (**C**). PTL group, region D, magnified image (**D**). PTU group, magnified image (PTU).

**Figure 7:**
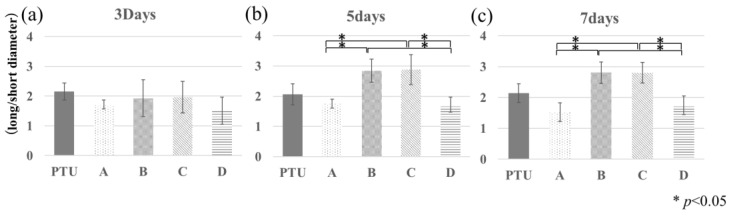
Bone lacunas morphometry. The vertical axis is ratio of long diameter to short diameter. Day 3, PTU and PTL groups (**a**). Day 5, PTU and PTL groups (**b**). Day 7, PTU and PTL groups (**c**). From day 5, compression is also evident within the compressed side, and a tendency becomes apparent for a division into areas where the bone lacunas are rounder and those where they are flatter.

Figure 6 shows images of polished specimens (Villanueva Osteochrome Bone Stain) from the PTU and PTL groups. In region A, the bone lacunas were round, and in region B, those in the PTL group were a flatter ellipse shape than those in the PTU group. On the uncompressed side, in region C, those in the PTL group were a flatter ellipse shape than those in the PTU group, and in region D, they were round. Figure 7 shows the results for the bone lacuna maximum long diameter/short diameter ratio. The bone lacunas in the normal rat femoral diaphysis showed an elliptical morphology with a ratio of long to short diameters of approximately 2:1, and the axial direction of the long diameter was nearly aligned with the direction of the external and internal periosteum (Figure 7a–c; PTU and PTL group). In regions A and D, the bone lacunas were rounder than those in the PTU group, with a ratio of approximately 3:2, and in regions B and C, they were flatter than those in the PTU group, with a ratio of approximately 3:1. The values in regions B and C were significantly higher than those in regions A and D.

**Figure 8:**
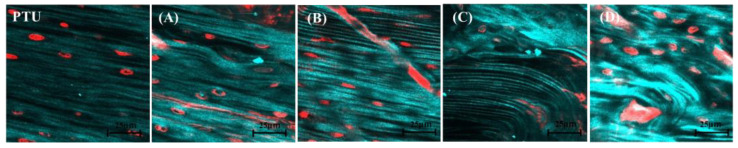
SHG imaging of the PTU and PTL groups. PTL group, region A (**A**). PTL group, region B, magnified image (**B**). PTL group, region C, magnified image (**C**). PTL group, region D, magnified image (**D**). PTU group, magnified image (PTU). Compared with the PTU group, the collagen fiber courses are disrupted in regions A and D and extended in regions B and C.

Figure 8 shows SHG images of the peri-implant bone of the polished specimens from the PTL and PTU groups. A layered structure of collagen fiber bundles parallel to the periosteum and endosteum was observed. This orientation was in the same direction as the long axis of the femur without the anchor screw implanted. However, in regions A and D, their courses were disrupted in a few places, and the collagen fibers appeared to be thicker in regions A and D and finer in regions B and C compared with the PTU group.

**Figure 9:**
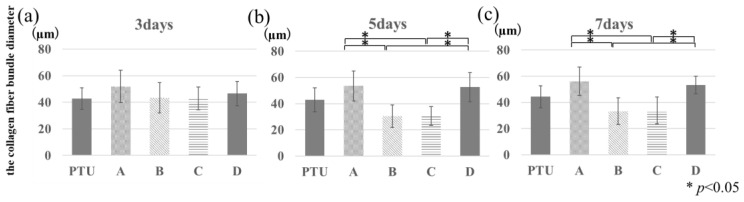
Collagen fiber bundle diameter measurements. The vertical axis is collagen fiber bundle diameter measurements (µm). Day 3, PTL and PTU groups (**a**). Day 5, PTU and PTL groups (**b**). Day 7, PTU and PTL groups (**c**). From day 5, the measured values for diameters tend to be higher in regions A and D than in regions B and C.

Figure 9 shows the collagen fiber bundle diameter measurement results. The diameter of collagen fiber bundles in normal rat femurs was approximately 40 µm. In regions A and D, they were thicker than those of normal femurs, and the values were significantly higher than those of regions B and C. Those in regions B and C tended to be finer than those of normal femurs. There was no significant difference with the PTU group in any of regions A, B, C, or D.

**Figure 10:**
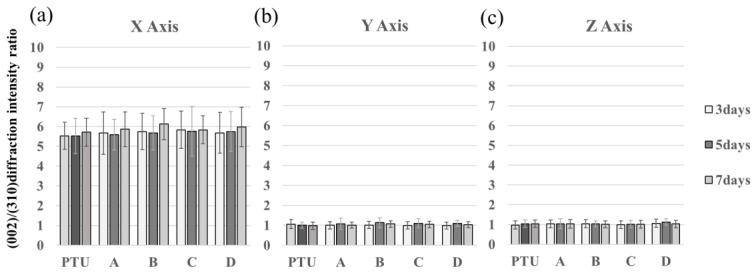
The vertical axis is (002)/(310) diffraction intensity ratio. X-ray diffraction intensity ratios in the PTU and PTL groups. X-axis diffraction intensity ratio (**a**). Y-axis diffraction intensity ratio (**b**). Z-axis diffraction intensity ratio (**c**). Although BAp crystal orientation in the direction of the X-axis (the long axis of the femur) was evident, this difference was not significant. There are no significant differences between day 3, day 5, and day 7.

Figure 10 shows the X-ray diffraction intensity ratios of the (002) and (310) planes in the measured regions in each group. The X-ray diffraction intensity ratio of hydroxyapatite (HAp) powder using transmission optics was 3.01, and that of HAP powder using reflection optics was 1.20. In all groups, a uniaxial preferential orientation in the X-axis direction was exhibited (Figure 10a). In the Y-axis and Z-axis directions, however, BAp crystals did not show a preferential orientation in any of the groups (Figure 10b,c). There was no significant difference between any of the groups.

## 4. Discussion

Orthodontic treatment using anchor screws enables the rapid achievement of reinforced anchoring by means of screws bonded to the bone as anchorage [23,24]. The biomechanical role of orthodontic anchor screws is very different from those of dental implants in that the former are never subjected to occlusal force loading but, instead, bear a large lateral load as an orthodontic force. Another major difference from dental implants, which are required to continue functioning in the mouth for as long as possible, is that it is assumed that anchor screws will be removed immediately after treatment [25,26]. In regular dental implant treatment, it is undoubtedly true that the lateral load must be minimized, but for orthodontic anchor screws, the lateral load is the main load, and they must, therefore, be adapted for a mechanical environment different from that of implants [20]. Implant surface treatment has already been shown to be effective for resisting occlusal force, but much remains unknown regarding its effect on the initial bone contact of screws placed under a lateral load [27].

Eriksson et al. investigated the effectiveness of surface treatment on the initial bone healing process. They reported that titanium that had been made hydrophilic exhibited better cellular reactivity during the first week and that blood clot formation also increased [28]. In the present study, the bone–implant contact rate (BIC) was significantly higher in the PTL and PTU groups on day 5 compared with the SUL and SUU groups. On day 7, however, both BIC and bone mineral density (BMD) had reached about the same level in the PTL, PTU, SUL, and SUU groups, with no significant differences. Lang et al. also described the effectiveness of hydrophilic modification on the bone contact rate, reporting that this increased at an earlier point in the surface treatment group but that this difference eventually disappeared [11]. In the present study, BMD was higher in the PTL and PTU groups compared with the SUL and SUU groups on day 3 and day 5, but not significantly. Similar to BIC, on day 7, the values were almost the same in all groups. These results showed that, although the surface treatment of screws could enable contact between anchor screws and bone to be achieved more quickly, the eventual contact rate was similar to that of screws that had not undergone surface treatment. This finding suggested that surface treatment might make the screw hydrophilic, improving cellular reactivity in the peri-implant jawbone immediately after insertion, promoting blood clot and osteoid formation and making it easier to achieve initial bone contact of screw immediately post-insertion.

On the compressed side, the bone lacunas were round in region A and elliptical in region B. On the uncompressed side, however, they were elliptical in region C and round in region D. The results of bone lacunas morphometry (the ratio of the long diameter to the short diameter) showed that this ratio was close to 1.0 in regions A and D and higher in regions B and C. Sims et al. investigated changes in cell morphology when cells were placed under mechanical stress, and they found that cells subjected to a contractile force exhibited roundish deformation. The results of the present study resembled those reported by Sims et al. [29]. Liu et al. reported that in a finite element analysis of the bone surrounding anchor screws subjected to lateral forces, compressive and tensile stresses were applied to both the compressed side and the uncompressed side, with the compressive stresses in the upper part of the compressed side and the lower part of the uncompressed side [30]. These results suggest that the lateral load imposed on the screws caused the screws to strain obliquely, mainly in the cortical bone, so that, on the compressed side, compressive stress was generated at the upper part of the screw and tensile stress at the lower part. Therefore, it was considered that the simple comparisons that have been conducted until now between the peri-implant bone on the compressed and uncompressed sides were inadequate, and instead, a comparative investigation was performed by carrying out a bone quality analysis of the upper (A) and lower (B) areas of the compressed side and the upper (C) and lower (D) areas of the uncompressed side.

The collagen fiber bundle diameter-measurement results showed that in regions A and D, the diameters tended to be larger because collagen fiber bundles were loose. In regions B and C, however, the tractive force applied tension to the collagen fiber bundles, causing them to extend and reducing their diameter. Martin et al. reported that the imposition of a tractive force causes collagen fibers to extend [31]. This result was similar to the present findings, suggesting that it may have been a reaction to the lateral load imposed on the screws.

BAp crystal orientation increased in regions A and D, but there was no significant difference between any of the groups. This may have been because the maximum observation time of 1 week was too short for sufficient remodeling to occur for changes in BAp crystal orientation to become evident. Nakano et al. reported that bone quality took longer to recover than bone density [32], and a preferential orientation of BAp crystals optimized for lateral load might only become evident after more time has elapsed. The same measurements were also performed for the screws that had not undergone surface treatment, but the results were not different from those of the surface treatment group, with no significant difference resulting from surface treatment.

The present findings suggest that screw surface treatment may speed up initial bone healing and make initial bone contact with screws easier to achieve. The pre-insertion surface treatment of anchor screws in orthodontic treatment is thus desirable to obtain anchorage as rapidly as possible. The application of a lateral load to these screws produces an oblique strain, imposing compressive stress in the neck area and tensile stress at the tip on the compressed side, and since the stress is concentrated in the neck area of the compressed side, which may reportedly increase the risk of screw loss [33].

## 5. Conclusions

The results of bone contact rate and bone mineral content indicated that there was no significant difference between the surface–treated and untreated groups in the long term. However, in orthodontic treatment where a short-term fixation source is desired, surface treatment of screws may accelerate initial bone remodeling and facilitate initial bone contact, suggesting that early loading may be possible in clinical practice.

The results of the bone lacuna morphometry and collagen fiber bundle dynamics confirmed that the bone tissue responds differently in different areas of the bone around the screw when lateral loading is applied. In the future, further progress should be made regarding bone quality, including trends in BAp crystal orientation, which did not respond in the short-term course.

## Data Availability

The data presented in this study are available on request from the corresponding author.
